# Aggressive hosts are undeterred by a cuckoo's hawk mimicry, but probably make good foster parents

**DOI:** 10.1098/rspb.2022.1506

**Published:** 2023-01-11

**Authors:** Mairenn C. Attwood, Jess Lund, Chima J. Nwaogu, Collins Moya, Claire N. Spottiswoode

**Affiliations:** ^1^ Department of Zoology, University of Cambridge, Cambridge CB2 3EJ, UK; ^2^ FitzPatrick Institute of African Ornithology, DST-NRF Centre of Excellence, University of Cape Town, Rondebosch, 7701 Cape Town, South Africa; ^3^ Musumanene Farm, PO Box 630303, Choma, Southern Province, Zambia

**Keywords:** brood parasitism, coevolution, cuckoo, mimicry, trade-off, aggression

## Abstract

Parasites face a trade-off if the highest quality hosts are also most resistant to exploitation. For brood parasites, well-defended host nests may be both harder to parasitize and harder to predate, leading to better survival of parasitic chicks. This trade-off could be accentuated if brood-parasitic adaptations to reduce front-line defences of hosts, such as mimicry of hawks by *Cuculus* cuckoos, do not deter hosts which aggressively mob raptors. Here we investigate the costs and benefits to the African cuckoo (*Cuculus gularis*) of specializing on a highly aggressive host species, the fork-tailed drongo (*Dicrurus adsimilis*). Field experiments showed that drongos strongly attacked and mobbed both cuckoo and hawk models, implying that hawk mimicry does not deter front-line defences against African cuckoos. Attacks on cuckoo and hawk models generally declined after the egg stage but attacks on snake models sharply increased, suggesting drongos may treat hawks more like cuckoos than predators. We suggest that the cost to cuckoos of parasitizing an aggressive host may be alleviated by subsequent benefits to their offspring, since drongo nests survived better than nests of other species with similar nesting ecology. These results are indicative of a trade-off between host quality and susceptibility for a brood parasite.

## 1. Introduction

Parasites face a trade-off if hosts which are more difficult to infect also improve the subsequent survival or transmission of the parasite [1–3]. High-quality hosts are likely to have greater defences against parasites, and therefore lower susceptibility to parasitism, yet represent a superior resource once a parasite is successful. There is evidence suggestive of such a trade-off across different forms of parasitism: for example, kleptoparasites steal food from either juveniles or adults, and while adults are better able to repel attacks, they typically yield larger prey items when successfully overcome [3,4]. Similarly, ectoparasites face stronger immune defences from higher resource hosts, but once successfully infected, such hosts allow greater parasitic fecundity [1,5]. Here we test whether brood parasites, which exploit the parental care of other species, experience such a trade-off—and ask what factors influence the magnitude of both costs and benefits.

For brood parasites, a trade-off could arise particularly when parasitizing highly aggressive hosts which may obstruct or injure a female attempting parasitism [6], but then subsequently improve the fecundity of successful parasites. This is because brood-parasitic chicks depend entirely on their foster parents for growth and survival, and so all else being equal, brood-parasitic parents should benefit from choosing high-quality hosts for their offspring [7–9]. However, the very properties which make hosts attractive foster parents may also make them more difficult to parasitize. In particular, many birds defend their nests from both predators and parasites using ‘front-line’ defences that include nest placement and architecture, nesting aggregations, cooperative breeding, and parental aggression [10–12]. In each instance, avian brood parasites which are able to broach these defences may reap the benefits of increased survival of their offspring through decreased nest predation. In this study, we focus on host aggression and mobbing as a front-line defence, which may make egg-laying more difficult for a parasite [6,13–16].

Host front-line defences have selected for reciprocal adaptations in parasites. These include rapid and sneaky egg-laying and, in many cuckoo species, mimicry of predatory bird species which deters hosts from attacking the cuckoo. For example, the common cuckoo (*Cuculus canorus*) mimics the Eurasian sparrowhawk (*Accipiter nisus*), which reduces mobbing by Eurasian reed warbler (*Acrocephalus scirpaceus*) hosts [17–19]. Hawk-like features are also shown by many other old-world cuckoo species and often specifically match local raptor species, suggestive of a widespread mimetic function of this phenotype [20]. However, studies of common cuckoo parasitism of more aggressive host species have found limited benefits of hawk mimicry in reducing attacks [21–23].

The African cuckoo (*Cuculus gularis*) closely resembles the common cuckoo, with which it shares features known to be important for hawk mimicry, including barred plumage and yellow eyes [20,24]. However, unlike the common cuckoo, this species almost exclusively parasitizes a host which readily attacks hawks, the fork-tailed drongo (*Dicrurus adsimilis*) [25,26] (hereafter ‘drongo’). Batesian mimicry, such as hawk mimicry by common cuckoos, relies on the host being deterred from interacting with raptors, but since drongos readily attack hawks [25,26], they may not be deterred from mobbing a hawk-like cuckoo. African cuckoo parasitism is particularly costly to drongos because, like other *Cuculus* species, the cuckoo hatchling ejects host eggs and chicks and monopolizes the nest; consequently, drongos have evolved sophisticated anti-parasitic defences including egg signatures to aid the detection of parasitic egg mimics [27,28]. Front-line defences would act in addition to these egg-stage defences, by reducing the number of parasitic eggs reaching this point. Even when eggs would be successfully rejected, front-line defences benefit drongos as the African cuckoo removes a drongo egg while laying [29].

To test the effectiveness of hawk mimicry by the African cuckoo, we can examine how a drongo perceives both hawks and cuckoos. While both threats are expected to elicit an aggressive response, we can exploit the fact that the importance of each threat varies over time: parasitism is only a risk early in nesting (cuckoos lay during or soon after the host's laying period, as parasitic eggs laid later are unlikely to be successful), whereas hawks are a threat to the adults throughout the nesting cycle [30], and an escalating threat to the brood as it increases in reproductive value with age [31,32]. A recent meta-analysis has shown that defences against parasites often decrease over the nesting period, while defences against predators either increase or remain constant [33]. Investigating how mobbing responses change across nesting stages can therefore reveal how a host perceives a threat: responses to a perceived parasitic threat should decrease over the nesting cycle, whereas responses to other threats should increase or remain constant.

In this study, we test two related hypotheses. First, we test whether fork-tailed drongo aggression undermines the effectiveness of hawk mimicry by African cuckoos. To do this, we use model presentations at drongo nests to test how drongos respond to hawks and cuckoos, with dove (harmless) and snake (nest predator) models as controls. Second, we test whether the costs African cuckoos face in parasitizing an aggressive host are offset through increased nest survival [27]. If this is the case, then drongos should experience higher nest survival than other open cup-nesting species breeding synchronously in the same habitat. The costs of aggression to cuckoos could be offset regardless of whether any higher nest survival is owing specifically to drongo aggression, or other differences in nesting ecology between plausible host species. If higher nest success is directly owing to drongo aggression, we further expect that the nests of more aggressive drongo pairs should experience higher nest survival than those of less aggressive pairs.

## 2. Methods

### (a) Study system and site

We carried out experiments during September–November in 2019 and 2021, on Semahwa, Musumanene and neighbouring farms (a matrix of miombo woodland and agriculture, centred on 16°45′ S, 26°54′ E) in the Choma District of Zambia. The drongo population here experiences a very high rate of parasitism by the African cuckoo; strong selection pressure on defences is therefore expected. Although cuckoo eggs or chicks were only found in 10% of 162 drongo nests in 2019, and in 7% of 128 nests in 2021, many cuckoo eggs were probably rejected before nests were found or between checks, as simulations based on field data estimate that 94% of cuckoo eggs are rejected at this site [27,28]).

### (b) Model presentation experiment

At 93 drongo nests in 2019, we presented models of three species: an African cuckoo, a little sparrowhawk (*Accipiter minullus*), and a ring-necked dove (*Streptopelia capicola*). For each species we used two different models, which experienced similar mobbing responses. All models were 28–29 cm in length, matching their natural sizes. Little sparrowhawks were selected as the model hawk species based on their similar size to African cuckoos, occurrence at the study site, and coloration; records indicate they predate both adult fork-tailed drongos and nests [34]. We selected ring-necked doves as the non-threatening control species because they are similar in size to both African cuckoos and little sparrowhawks, and common at the field site. Models were three-dimensionally printed in polylactic acid and painted with acrylics (see the electronic supplementary material, S1); previous studies have shown painted models are treated similarly to taxidermic mounts and real birds [13,19].

At 63 drongo nests in 2021, we again presented models of three species: a rubber snake (Supkeyer 130 cm), similar in size and appearance to a boomslang *Dispholidus typus* (a common predator of birds’ nests at this site), a little sparrowhawk, and a ring-necked dove (cuckoo presentations were not repeated owing to time and habituation constraints when presenting four models on the same day). One set of experimental presentations was carried out per drongo pair per year. It is possible that some drongo pairs were used for different experiments in both 2019 and 2021, but we minimized pseudoreplication by identifying and avoiding pairs nesting at the same location and with the same egg signature phenotype as a pair two years before.

The models were presented in a random order with an hour between trials. Prior to each trial, we set up a video recorder (Sony CX280 Full HD Handycam) in a blind 10 m from the nest and tied the model to a branch approximately 2 m from the nest. Trials were filmed and lasted for 5 minutes, beginning when a drongo came within 5 m of the model. Trials were abandoned if no drongo appeared in the 10 minutes after the model was set up (*n* = 31).

### (c) Quantifying drongo responses

We quantified mobbing dives towards each model from video recordings. Mobs were defined as close swoops accompanied by a short sound (see the electronic supplementary material, video S1), and sometimes involved direct contact with the model. As it was not possible to count blind to treatment type, mobs in a subset of videos were independently counted by another observer without prior knowledge of the experiment. Mobbing counts were highly repeatable across observers (rptR package [35] using the generalized linear mixed model (GLMM) method and log link, *n* = 21, *R* > 0.99, *p* < 0.001).

We also examined the subsequent nest attentiveness of drongos (a proxy for parasitism-specific defence [15,36,37]), since hawk mimicry could avoid giving drongos a cue of parasitism, and thereby reduce rejection of cuckoo eggs, even if it does not deter drongos from mobbing. In such a scenario, hawk mimicry would be adaptive to cuckoos via aggressive rather than Batesian mimicry (i.e. mimicry of a ‘harmless’ model with respect to parasitism risk) [38]. However, neither metric of nest attentiveness (time spent at nest or number of times looking at the clutch) differed between the dove control and cuckoo, suggesting that neither was a useful proxy for drongo perception of parasitism, precluding a specific test of aggressive mimicry (electronic supplementary material, S2).

### (d) Analysis of model presentation experiment

All analyses were conducted in R v.4.1.3 [39]. To compare responses to different models, we constructed a negative binomial GLMM with the lme4 package, as responses were over-dispersed count data and therefore not normally distributed [40]. We included nest identity as a random term to account for the presentation of multiple models at each nest. We examined correlation between fixed effects for each model and found no evidence for collinearity.

Model selection was performed via an information criterion (Akaike information criterion; AIC) approach [41,42], using the MuMIn package to calculate AIC_c_ values for biologically plausible combinations of parameters [43]. The global model included the following covariates as fixed effects: an interaction term between model species and age of nest, the order in which the model was presented (first, second or third, coded as an ordinal variable), hours after sunrise (continuous) and the year (categorical). An additional covariate of Julian date within the season was coded as a random effect. The model also included an offset for the total number of ‘drongo seconds’ over which mobbing occurred (300–600, depending on the number of adults present over the 5 min trial; for details on whether one or two drongos attended model presentations, see the electronic supplementary material, S3). The power to detect the smaller of two standardized effect sizes reported by a comparable study (for differences in host responses to common cuckoo and hawk models, *d* = 0.77) was greater than 0.99 in this study, suggesting that we had ample power to detect a difference if there was one. We present the coefficients for the top model by AIC in the results, as only this model had ΔAIC < 2.

### (e) Comparing nest survival across species

We compared the nest survival of drongos (*n* = 226) with dark-capped bulbuls *Pycnonotus tricolor* (*n* = 85) and tchagra bush-shrikes (the ecologically similar brown-crowned tchagra *Tchagra australis* and black-crowned tchagra *Tchagra senegalus*; *n* = 75). These species use open cup nests to breed in similar habitats and at similar times of year and thus have comparable nesting ecology to drongos [34]. There are no records of parasitism at the study site from either our fieldwork or a historical dataset (*n* = 197 nests for bulbuls, and *n* = 291 nests for tchagras). Our assumption that drongos are more aggressive than these species was supported by experiments in which we presented hawk models for 5 minutes (from the adults’ return) at nests with late-stage eggs or young chicks between 29 September and 10 October 2022. Bulbuls (*n* = 10) and tchagras (brown-crowned, *n* = 7; black-crowned, *n* = 3) did not mob the models during any of the trials; their most common response was to give alarm calls from *ca* 5 to 10 m away. Drongos (*n* = 10) mobbed the hawk model in 7 of the 10 trials (number of mobbing dives ranging from 6 to 115). One trial was cut short after 2 minutes because drongo mobbing decapitated the hawk model.

We monitored drongo and bulbul nests in 2019, and nests of all species in 2021. We checked nests for predation every 3 days, ±1 day. We then investigated whether species predicted nest survival, using a semi-parametric Cox proportional-hazards model in the R package ‘survival’ [44]. Time was modelled continuously, with predation assumed to occur at the midpoint between the last day where the clutch was known to be present and first known to be predated (inferred when the nest was found empty, prior to fledging).

The data were left-truncated because clutch initiation often started before observations, which began when the nests were found. This was accounted for by including an estimated date of clutch initiation. Data were right-censored if chicks fledged, if there was human interference, or if visits ceased before predation/fledging. We tested the assumption of hazards being proportional (i.e. not varying differentially across predictors with time) by using the cox.zph function to perform Schoenfeld tests and plot residuals. Year and Julian date within the season at which nesting commenced were included as additional predictors [45–47]. We used the same information criterion approach for model selection as described above and present the best model by AIC.

For an intuitive sense of survival rates, we also used the Mayfield method on these data. This calculates daily survival rate as 1 – (number of failed nests/total number of exposure days) and overall survival rate as this value to the power of the average nesting length (in days) [48,49].

### (f) Drongo aggression as a predictor of nest survival

We further tested whether any differences in drongo nest survival were correlated with a drongo pair's aggression levels (*n* = 78 nests in 2019 and *n* = 63 in 2021, for which there were also aggression data). The subset of nests we followed was dictated by time constraints and therefore probably random with respect to predation risk. To obtain nest-specific estimates of aggression, we used the random intercept coefficients from the GLMM described under ‘Analysis of model presentation experiment’. These estimates reflect consistent variation in absolute mobbing intensity between nests that could not be explained by other factors.

We used the same survival analysis approach as described for the between-species data, again with year and Julian date as additional predictors. Data were right-censored if chicks fledged (*n* = 59), if there was human interference (e.g. eggs collected for another study, *n* = 11), or if visits ceased before predation/fledging (*n* = 30). Nest height was not a significant predictor of survival in nests for which it was measured (all those in 2021; *n* = 62, *β*_coefficient_ = 0.09, *z* = 0.927, *p* = 0.35).

For both the intra- and inter-specific survival comparisons, we analysed the survival of chicks in their natal nests. Survival of parasitic chicks in the same nests may differ (e.g. owing to conspicuousness in the nest), but there were too few cuckoo chicks to test this directly. Instead, we assume that the impact of parental defences on nest survival would have the same directionality for both host and parasitic chicks.

## 3. Results

### (a) Are there costs of parasitizing an aggressive host?

**Figure 1.**
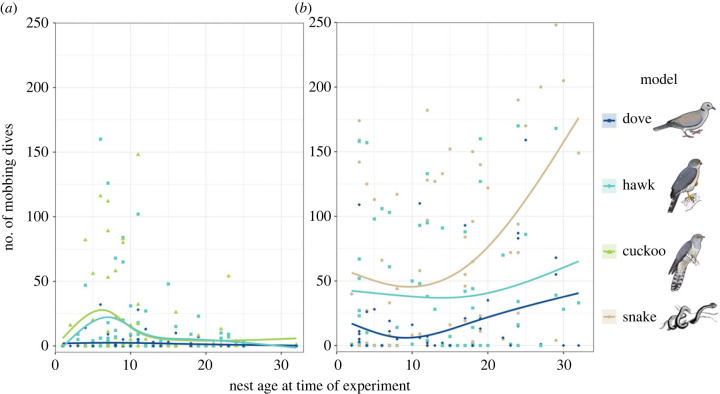
Total number of mobbing dives in response to different models over the drongo nesting cycle. (*a*) In 2019, cuckoo, hawk and dove models were presented. (*b*) In 2021, snake, hawk and dove models were presented. Nest age is counted from the first day of incubation (after clutch completion). Points show the data from individual trials, with smoothed lines added using the ‘glm’ method. The statistical analysis (table 1) confirmed that aggression was high towards the cuckoo, hawk and snake models but not towards the dove control, and that aggression declined across nesting stages for both cuckoo and hawk models. Bird illustrations reproduced with permission from Faansie Peacock | FIREFINCH.

Drongos responded to models with repeated mobbing and alarm calls. In 2019, hawk and cuckoo models were mobbed most intensively at the start of nesting, each to a similar degree (figure 1*a*), while dove models received low levels of mobbing throughout. Results differed between years, with mobbing higher in 2021 across all treatments, including the dove control (figure 1*b* and table 1). Cuckoo models were not presented in 2021 (see Methods), precluding comparison of responses to hawks and cuckoos in this year. Aggression towards hawk and cuckoo models varied similarly over nesting stages, since the interaction between the cuckoo model and nest age was significantly different to that for the dove model and snake model, but not the hawk model (table 1). The GLMM showed that overall across years, responses to both cuckoo and hawk models decreased across nesting stages (table 1), but visual inspection of the data suggests that for hawks this was less pronounced in 2021 than 2019 (figure 1). However, responses to hawk models contrasted with those to snake models presented in 2021, which strongly increased across nesting stages (figure 1, table 1). Aggression declined with the order in which models were presented, suggesting either habituation or fatigue in responses to the later-presented models. Time of day did not appear in the top models (by AIC; electronic supplementary material, S4), and Julian date within the season was not significant in Wald's *χ*^2^ tests of coefficients from the model-averaged summary (table 1). Inclusion of the random term (nest identity) was important for model fit, based on AICc.

**Table 1.** The coefficients for the top negative binomial GLMM by AIC, showing the effect sizes and *p*-values for predictors of drongo mobbing intensity (d.f. = 14). (There was a significant interaction between model species and nest age, with responses towards doves and snakes (but not hawks) over nesting differing to those towards cuckoos. Year and order of model presentation were also significant predictors of mobbing response. *p<0.05; **p<0.01; ***p<0.001.)

	estimate ± s.e.	*z*-value	Pr(>|*z*|)
intercept	−4.62 ± 0.54	−8.57	<0.001***
nest age	−0.07 ± 0.04	−1.93	0.05
dove versus cuckoo model	−2.66 ± 0.56	−4.77	<0.001***
hawk versus cuckoo model	−0.78 ± 0.52	−1.49	0.137
snake versus cuckoo model	−1.20 ± 0.69	−1.73	0.08
presentation order (linear)	−0.65 ± 0.16	−4.16	<0.001***
presentation order (quadratic)	0.26 ± 0.15	1.80	0.07
year (2021 versus 2019)	2.07 ± 0.37	5.64	<0.001***
dove × nest age versus cuckoo × nest age	0.11 ± 0.04	2.62	0.01*
hawk × nest age versus cuckoo × nest age	0.06 ± 0.04	1.65	0.10
snake × nest age versus cuckoo × nest age	0.14 ± 0.05	3.04	<0.01**

In summary, aggression was high towards the cuckoo, hawk and snake models but not towards the dove control, suggesting that hawk mimicry does not deter highly aggressive drongos. Aggression towards cuckoo and hawk models decreased overall across nesting stages, whereas aggression towards the snake model strongly increased.

### (b) Are there benefits to parasitizing an aggressive host?

**Figure 2.**
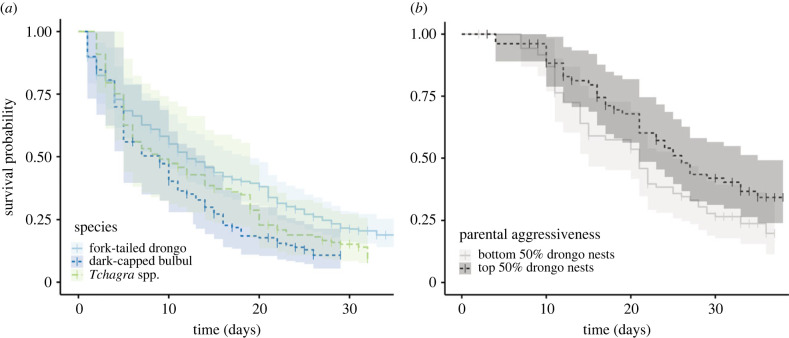
Kaplan–Meier survival curves for nesting birds, combining data from 2019 and 2021. Shaded areas show 95% confidence intervals. (*a*) Survival compared across species with comparable nesting ecology; fork-tailed drongo nests had significantly higher rates of survival, compared to dark-capped bulbuls (beta coefficient = 0.59, *p* < 0.001) and tchagras (beta coefficient = 0.51, *p* < 0.01). (*b*) Survival for drongo nests in relation to parental aggression. For this visualization, we split the nests based on parental aggression, with the top 50% shown in black and the bottom 50% in grey. Parental aggression was taken as the coefficients of random intercepts in a model on the drongo mobbing rate of both hawk and cuckoo models (Methods). For statistical analyses, aggression data were treated as continuous, and we found no effect of parental aggression on survival probability (this predictor did not feature in the top models, based on AIC).

First, we compared the nest survival of drongos to that of other open cup-nesting species breeding syntopically. We found significant differences in survival between drongos, dark-capped bulbuls and tchagras (figure 2*a*). Drongo nests showed higher rates of survival than both bulbuls (bulbuls compared to drongos *β*_coefficient_ = 0.59, *z* = 3.49, *p* < 0.001) and tchagras (tchagras compared to drongos *β*_coefficient_ = 0.51, *z* = 2.73, *p* < 0.01). From Mayfield's estimates, overall drongo nest survival was 20.2% (daily survival rate (DSR) = 0.953, average nesting length 33 days); bulbul nest survival was 9.8% (DSR = 0.92, average nesting length 28 days); and tchagra nest survival was 13.7% (DSR = 0.936, average nesting length 30 days).

Second, we analysed nest survival across drongo pairs. Parental aggression did not appear in the top model based on AIC (electronic supplementary material, S4) and was not a significant predictor of drongo nest survival in the full model (figure 2*b*). Nesting date within the season also did not appear in the top model based on AIC, with year the only covariate to do so (with a trend towards higher survival in 2021, although this was not significant in the top model; *β*_coefficient_ = −0.34, *z* = −1.55, *p* = 0.12).

## 4. Discussion

These results show no mimetic advantage to the African cuckoo's hawk-like appearance in their interaction with fork-tailed drongos. Drongos responded in the same way to hawk and cuckoo models when both were presented at nests in 2019, and their aggression towards both model types decreased in a similar way over nesting stages. Since cuckoos are only a threat to their hosts early in the nesting cycle, whereas hawks impose the greatest fitness costs on hosts later in the nesting cycle, these findings suggest that drongos may have treated hawks as cuckoos in 2019, rather than vice versa. If this is generally true, it would stand in contrast with other systems in which hosts distinguish the two, and/or err on the side of treating cuckoos as hawks [17,21,23].

All models were mobbed less strongly in 2019 than 2021, perhaps owing to low resource conditions in 2019, when the breeding season was delayed and followed a drought throughout the region [50]. In 2021, greater resource availability may have enabled greater levels of aggression towards all species in close proximity to nests, although we did not directly test how drongos responded to cuckoos in that season. Social learning of nest defences may rapidly promote different responses by hosts, dependent on the conditions in a particular year [51–53]. Nonetheless, the significantly greater increase in drongo aggression towards snakes than towards hawks over the nesting cycle in 2021 suggests that hawks are treated distinctly from a nest predator.

We found some evidence that the costs to cuckoos of high aggression early in the nesting cycle could be counterbalanced by survival benefits for parasitic offspring. Cuckoos may benefit from parasitizing drongos rather than other species, as drongo nests survived better than ecologically similar dark-capped bulbuls and tchagras (figure 2*a*). However, there was no evidence that, among drongos, more aggressive pairs had better surviving nests (figure 2*b*). It is therefore unclear whether the higher survival of drongo nests at the species level is owing to parental aggression, and/or to other differences in nesting ecology. In either case, higher survival of drongo nests should increase the proportion of cuckoo chicks raised to fledging once a cuckoo has successfully broached drongo anti-parasitic defences.

Why did drongos treat both cuckoos and hawks in the same way? Either drongos adaptively mob each species independently to the same degree, or they do not discriminate between the two. If the latter, this would contrast with multiple other brood-parasitic systems where hosts distinguish other cuckoo species from the local raptor species they resemble [17,21,23,54–57]. It is likely that for those hosts, interacting with a hawk represents a greater risk than it does for a drongo, a species which regularly responds aggressively to raptors [25]. For drongos, the fitness cost of mistakenly treating hawks as cuckoos may be lower than risking parasitism from the reverse error, particularly when resources are limited. If so then it would be adaptive for drongos to treat hawks as cuckoos if they are unable to confidently distinguish between them. More abundant resources may permit elevated aggression to all similar threats, which might explain the stronger absolute response to hawk models in 2021 than in 2019. Further experiments outside the breeding season would be useful to ascertain whether drongo responses differ when the costs of recognition errors change.

More broadly, there are relatively few other deceptive mimicry systems in which a receiver appears to treat both model and mimic as the mimic. This may be partly because mimics do not benefit in this situation, so there is no selection to maintain the mimetic phenotype. We would therefore expect this situation to arise only if the mimetic phenotype initially evolved in a context where it was beneficial and carries insufficient costs to be selected against. A comparable situation is the currently unsuccessful aggressive mimicry of female southern red bishops (*Euplectes orix*) (a harmless non-parasitic bird) by brood-parasitic cuckoo finches (*Anomalospiza imberbis*). Here, both harmless models and parasitic mimics elicit equal (and elevated compared to controls) mobbing responses by tawny-flanked prinia (*Prinia subflava*) hosts [58]. Mobbing southern red bishops incurs primarily an energetic cost, which is likely low relative to risking parasitism. The same balance of trade-offs would be more surprising in the African cuckoo–drongo system, where mobbing a hawk presumably entails greater risk.

The absence of a clear benefit from hawk mimicry to the African cuckoo may also explain why the African cuckoo lacks an adult rufous morph [59], despite the co-occurrence of plausible raptor models across parts of its range (e.g. rufous-chested sparrowhawk *Accipiter rufiventris* and rock kestrel *Falco rupicolus*). In the closely related common cuckoo, it is possible that a polymorphism between grey and rufous plumage types impede recognition of the mimic by some hosts [60,61]; but see [62]. Where hosts have the potential to distinguish the model and mimic, alternative morphs can limit learning opportunities. However, given that drongos did not distinguish between model and mimic, there may be no selective pressure to diversify mimetic phenotypes and maintain multiple cuckoo morphs.

Why do African cuckoos resemble hawks? This is puzzling, since African cuckoos seem to gain no advantage from their hawk-like appearance in their interactions with nesting drongos. In the common cuckoo, mimicry may be maintained owing to success in at least some hosts, whereas African cuckoos specialize almost exclusively on drongos [26]. There may be no adaptive benefit, such that this phenotype is either selectively neutral or a case of evolutionary lag. Under such a scenario, hawk mimicry may have originated ancestrally in the context of a different host, given that hawk-like features are prevalent across most of the genus *Cuculus* [59,63,64]. However, hawk-like plumage patterns appear to respond to selection over comparably short evolutionary timespans in other cuckoo species [20], casting doubt on evolutionary lag as an explanation. Alternatively, these features may confer an adaptive advantage in other situations, or for reasons aside from mimicry. Even where hawk mimicry is confirmed to aid parasitism, it may have additional functions such as protecting against raptor attacks, or improving crypsis [17,63].

Given these apparent costs of parasitizing an aggressive host, why do African cuckoos specialize almost exclusively on parasitizing drongos? Our species-level analysis supported the hypothesis that cuckoo offspring benefit from the relatively high survival of drongo nests. This suggests that offspring of cuckoos which lay their egg successfully in drongo nests are more likely to avoid nest predation than if they had been laid in nests of other co-occurring species. It is plausible that this benefit arises specifically from the high aggression of drongo parents, but we cannot be certain of this with our correlational data. We found that at the level of individual drongo nests, survival was unrelated to the drongo parents’ level of aggression to threats at the nest, suggesting that cuckoos would not benefit from selectively parasitizing the best-defended drongo nests. A possible explanation is that conspicuous defences draw the attention of predators towards nests [65–69]. If true, this may compound selection for speedy egg-laying by brood parasites, in addition to the benefits of avoiding physical harm and decreasing the likelihood of egg rejection [70,71]. Alternatively, factors which affect both nest survival and aggression may have confounded our correlational data (e.g. nest exposure may cause both higher aggression and lower survival). Drongo aggression levels may also be consistently high from a predator's perspective, such that intra-species variation is of little relevance to a predator [66]. Finally, it is possible that there is a small effect of intraspecific aggression on nest survival, which we did not have the power to detect in this study.

## 5. Conclusion

This study found no benefit of hawk mimicry to the African cuckoo, since its fork-tailed drongo hosts strongly attacked both hawk and cuckoo models. The hawk-like plumage of African cuckoos may thus either confer no adaptive advantage, or bring benefits in contexts outside of parasitism. Cuckoos thus appeared to experience a cost of parasitizing a highly aggressive host species, which their hawk mimicry was unable to mitigate. This cost to cuckoos is weighed against the potential fitness benefits of improved offspring survival in drongo nests compared to nests of other similar species. This study thus supports the hypothesis of a trade-off between host quality and susceptibility in a brood-parasitic system. Understanding the extent to which this trade-off is found across parasitic and predatory interactions will help us to explain the diversity of outcomes of antagonistic species interactions.

## Data Availability

Data are available from the Dryad Digital Repository: https://doi.org/10.5061/dryad.gb5mkkwsh [72]. Additional data are provided in the electronic supplementary material [73].
